# Establishing the criterion validity of an adapted dietary screener for Asian Americans amongst Chinese American adults

**DOI:** 10.1186/s13690-023-01158-4

**Published:** 2023-08-11

**Authors:** Lena Woo, Stella S. Yi, Agnes Park, Lu Hu, Lorna E. Thorpe, Pasquale E. Rummo, Jeannette M. Beasley

**Affiliations:** 1 Renaissance School of Medicine, Stony Brook University, Stony Brook, NY USA; 2 Department of Population Health, NYU Grossman School of Medicine, New York, NY USA; 3 Department of Medicine, NYU Grossman School of Medicine, New York, NY USA; 4 Department of Nutrition and Food Studies, NYU Steinhardt School of Culture, Education, and Human Development, New York, NY USA

**Keywords:** Dietary screener, Chinese american adults (CHAs), Criterion validity, Diet assessment, ASA-24

## Abstract

**Objective:**

To assess the criterion validity of a dietary screener questionnaire adapted for Asian Americans (ADSQ) compared to Automated Self-Administered 24-Hour Dietary Assessment Tool (ASA-24) food diary data amongst Chinese American Adults (CHAs). The ADSQ incorporated example ethnic foods from six Asian American groups. Lessons learned with respect to translating the ADSQ from English into Simplified Chinese were also documented.

**Design:**

Agreement between a two-day food diary (one weekend day and one weekday) and the ADSQ was assessed for vegetable, fruit, dairy, added sugar, fiber, calcium, and whole grain intake using paired t-tests to compare means and Spearman correlations to assess agreement between intake of food components.

**Setting:**

Data were collected online and via phone interviews.

**Participants:**

Thirty-three CHAs aged 19–62 years (63.6% female).

**Results:**

Mean differences were small for fruit, dairy, fiber, calcium, and whole grain intake, but were significantly different for vegetables and added sugar intake. Spearman correlations were < 0.5 and non-significant (p > 0.05) for all components. Both the ASA-24 and the ADSQ identified the same categories where CHAs intake is misaligned with dietary recommendations: whole grains, total fruit, and dairy. Difficulties were encountered in translating 13 out of 26 questions.

**Conclusions:**

The ADSQ may be a useful tool to identify intervention targets for improving dietary quality, but caution is warranted when interpreting vegetable and added sugar estimates. Differences in the English and Chinese languages underscore the need to take into account both literal translations and semantics in translating the ADSQ into other languages.

**Table:** 

**Text box 1. Contributions to the literature**
• Asian Americans consume a wide variety of foods, and consumption varies widely amongst Asian American subpopulations. Most ethnic foods are not included on American dietary questionnaires, which is a barrier to effective research of Asian American dietary habits.
• There is little research on Asian American diets, especially when compared to those of non-Hispanic Whites, Hispanic Whites, and Black Americans, which necessitates the creation of a screening tool specific for Asian Americans.
• To date, the Adapted Dietary Screener Questionnaire (ADSQ) is the only dietary questionnaire made for use amongst Asian Americans.
• Validating this dietary screener for use amongst Chinese Americans is a first step in moving to gain more information on a subgroup of Asian American diets and ultimately decrease disparities.

## Introduction

Asian Americans are the fastest-growing racial group in the United States, with 24 million people (roughly 7.2% of the total population) identifying as Asian American in 2020 [1, 2].

However, little is known about their health and health behaviors, due to a lack of funding to study health and disease in Asian Americans that has been recognized by the National Institutes of Health [3, 4]. While Asian Americans have historically been perceived as healthier than other racial/ethnic groups [5, 6], studies conducted in this population indicate otherwise. Data from the 2011–2016 National Health and Nutrition Examination Survey (NHANES) revealed that only 15.9% of US-born Asian Americans aged 20 years and older had ideal cardiovascular health, which was measured using a composite score of adiposity, total cholesterol, blood pressure, blood glucose, smoking, physical activity, and diet. [7] Total diabetes prevalence within the non-Hispanic Asian group as a whole was 19.1%, compared to 12.1% for the non-Hispanic white group, and varied among subgroups: 14.0% for the East Asian subgroup, 23.3% for the South Asian subgroup, and 22.4% for the Southeast Asian subgroup [8]. A study examining disaggregated data for six Asian American subgroups (Asian Indian, Chinese, Filipino, Japanese, Korean, Vietnamese) similarly reported that these subgroups had significantly higher prevalence of type 2 diabetes compared to non-Hispanic whites, despite having a 5–8% higher treatment rate [9]. Data also suggest that weight gain may pose higher risks for Asian Americans than it does for their non-Hispanic white counterparts, which has led to organizations such as the World Health Organization to suggest, and for the American Diabetes Association to adopt, lowering the BMI cutoff (22–25 kg/m^2^ for observed risk, 25–28 kg/m^2^ for high risk vs. 30 kg/m^2^) for cardiovascular disease and diabetes mellitus risk for that population [10, 11].

It is well established that poor diet largely contributes to deaths from lifestyle-related diseases such as cardiovascular disease [12–14]. Dietary intervention is an effective strategy to control cardiovascular disease risk factors and reduce overall cardiovascular disease risk. However, currently available evidenced-based curricula for nutrition are not culturally tailored to meet the needs of the Asian American population [15], or more specifically, the Chinese American population. Development of dietary interventions and programs for this group is hindered by the lack of instruments to assess their unique dietary patterns. Use of current instruments may result in under- or over-estimation of nutrient intake from certain ethnic foods whose nutrient profile may be incorrectly estimated in non-adapted instruments [16, 17]. When these intakes are associated with disease outcomes, inaccurate correlations may exist between diet and disease risk profile, which therefore renders dietary recommendations inaccurate. Thus, there is a need for tailored instruments to capture the unique food group consumption of Asian Americans, as measurement error in dietary assessment impedes efforts to improve dietary intake among Asian Americans. Recognizing the need for such tools, co-authors adapted the NHANES Dietary Screener Questionnaire (DSQ) by adding culturally specific food items for six of the largest Asian subgroups in the US [18], which are Chinese, South Asian, Filipino, Korean, Vietnamese, and Japanese. The purpose of the Validating a Dietary Assessment study was to establish the criterion validity, defined as an index of how well a measure correlated with an established standard of comparison, of the culturally adapted DSQ (ADSQ) as compared to Automated Self-Administered 24-Hour Dietary Assessment Tool (ASA-24) [19] food record data. We hypothesized mean food category and nutrient estimates derived from the two dietary assessment tools would be similar, and that the ADSQ values would be directly correlated with those from the ASA-24 [20]. We also documented implementation lessons learned in translating the ADSQ from English to Simplified Chinese.

## Materials and methods

### Study design

Chinese-American participants (n = 33) were recruited via community-based outreach by multilingual (English, Mandarin, Cantonese) study team members and ResearchMatch, a non-profit program funded by the NIH that connects interested participants with research studies. Potential participants completed a screening questionnaire on Open REDCap. Eligibility criteria included being 18 + years old, the ability to read and speak English or Mandarin, self-identifying as Chinese-American, and having access to a personal computer, laptop, or tablet.

Individuals who were both interested and eligible for the study were sent in-language emails with study information and a URL to complete two questionnaires assessing dietary intake on REDCap: (1) the ADSQ, adapted from the NHANES DSQ for the six largest Asian American subgroups (Chinese, Japanese, Korean, Vietnamese, Filipino, Asian Indian), and (2) a dietary risk screener, as reported on elsewhere [21]. Within two weeks of completing the first two dietary assessment questionnaires, participants submitted food records for a weekday and a weekend day of their choosing. Participants were provided with food record templates with instructions on how to complete them, as well as a food measurements guide. Food, drinks, and supplements were self-reported and described in detail, as well as time consumed, location consumed, and portion size. Participants emailed this data to community health workers (CHWs), who then manually entered the data into the ASA-24, which is an automated tool developed by the National Cancer Institute that automatically codes 24-hour dietary recalls and single or multi-day food records [19]. CHWs clarified information from the food record from participants during short phone interviews. CHWs entered the data into the ASA-24 instead of participants due to previous experience indicating that it may be difficult for non-native English speakers or those with low technology literacy to navigate the ASA-24 platform [22]. Following completion of the dietary assessments, participants received a $30 Amazon gift card. All participants provided electronic informed consent, and the study was approved by the NYU Grossman School of Medicine Institutional Review Board.

### Translating into chinese

In our experience in working with the NYC Chinese American Adult (CHA) community, translations performed and reviewed by CHWs (vs. a translation company) are more acceptable to survey participants. Translation of the ADSQ from English into simplified Chinese required several iterations [see supplementary material for questionnaire in both languages]. First, a bilingual CHW translated it and emailed it to a second CHW. The second CHW reviewed it, and then sent it back with comments to the first CHW. The first CHW reviewed these comments, updated the translation, and sent it to two more bilingual study team members. Upon receiving their comments, the first CHW finalized the document for use in the study.

### Nutrient consumption

ASA-24 data were cleaned per procedures recommended by the National Cancer Institute (NCI), from which Healthy Eating Index (HEI)-2015 scores – a measure of diet quality as compared to the Dietary Guidelines for Americans [23]- were derived. Scores from days 1 and 2 (weekday and weekend) were averaged. For the ADSQ, scoring algorithms from the NCI were used to convert responses into dietary intakes of different components. Components include fruits and vegetables (cup equivalents), dairy (cup equivalents), added sugars (tsp), whole grains (ounce equivalents), fiber (grams), and calcium (mg). The added component of seafood is not in the original algorithm, so it was not included in the comparison. Paired t-tests were used to compare means of estimated intake reported via ADSQ and ASA-24.

### Correlations between ASA-24 and ADSQ scores

HEI-2015 food groups consistent with ADSQ food groups were compared. These included: total vegetables, total fruits, dairy, added sugar, fiber, calcium, and whole grains. Correlational analyses between amounts of nutrient intake were run using Spearman correlation coefficients. A two-sided alpha of 0.05 was used as a threshold for statistical significance. A sample size of 33 was sufficient to detect a correlation of 0.5 between the ADSQ and 24-hour food record results at 80% power at a statistical significance level of 0.05. Data were analyzed using SAS (Version 9, SAS Institute, Cary, NC), and SPSS (Version 28).

## Results

### Demographics

The sample was 63.6% female, 48.5% spoke English only, 12.1% spoke Mandarin only, and 39.4% spoke both (Table 1).

**Table 1:** Demographic Characteristics of Chinese American Participants (n = 33) in the Validating a Dietary Assessment study, 2021

	Frequency	Proportion
Gender Male Female	12 21	36.4 63.6
Age 19–25 26–35 36–45 46–55 56+	4 8 10 7 4	12.1 24.3 30.3 21.2 12.1
Language English Only Mandarin Only English and Mandarin	16 4 13	48.5 12.1 39.4

### Implementation lessons learned from translation from english into simplified Chinese

**Fig. 1:**
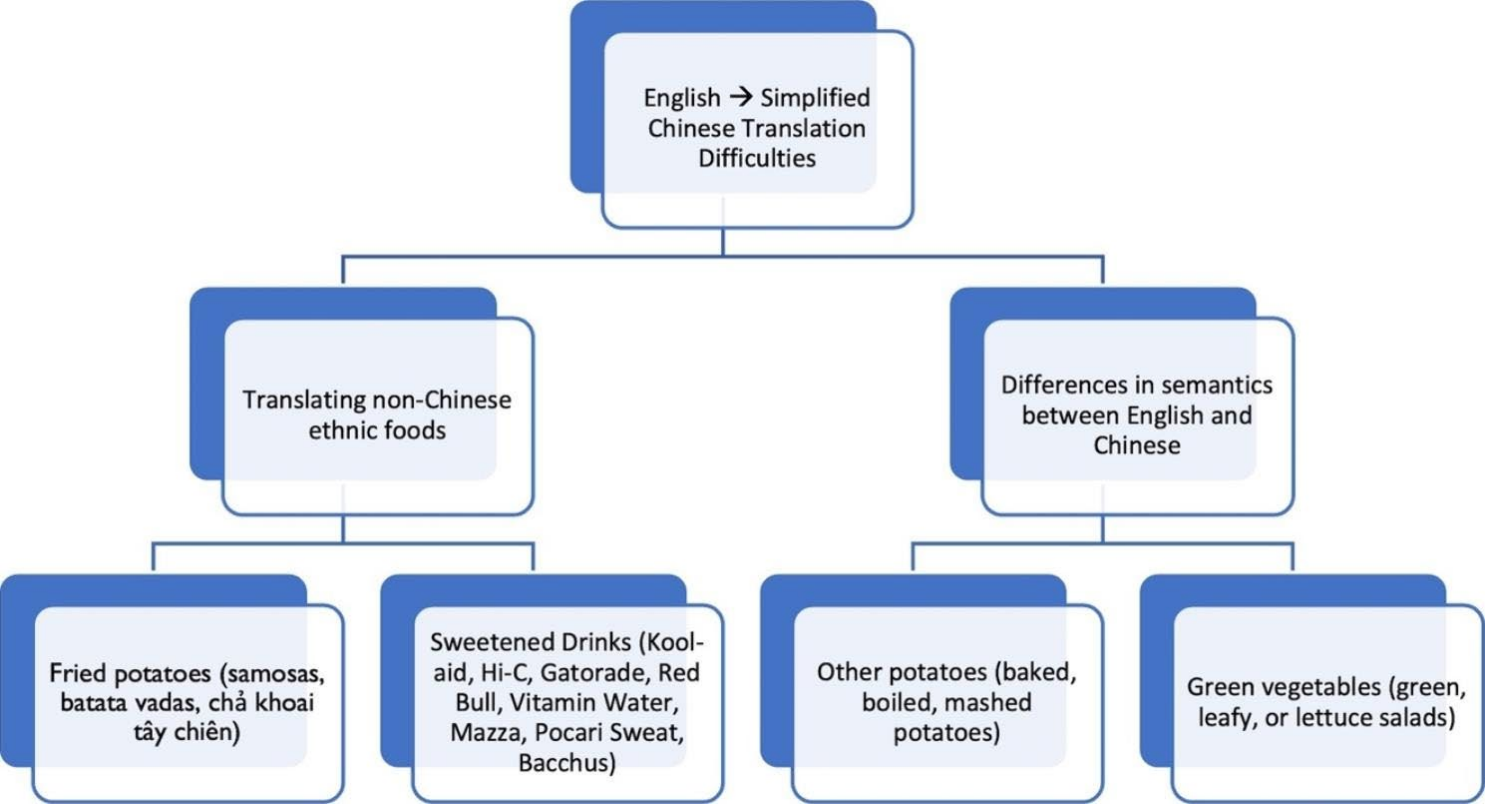
Translation Difficulties in the Validating a Dietary Assessment study, 2021

Of the 26 questions on the ADSQ, thirteen were easily translated into Chinese. These included questions that asked about fruits, vegetables, cooked whole grain items, pizza, red meat, seafood, whole grain bread, milk/yogurt/lactose-free milk, regular soda, 100% pure fruit juices, chocolate and candy, ice cream/frozen desserts, and popcorn. Difficulties were encountered in translating the remaining half of the questions. Difficulties can be summarized into two categories: (1) difficulties translating non-Chinese ethnic foods and (2) differences in semantics between English and Chinese (Fig. 1).

Difficulties translating non-Chinese ethnic foods: Seven questions contained non-Chinese ethnic foods that were difficult to translate. Two of them contained potato-based items, such as samosas, batata verdes, chả khoai tây chiên and aloo sabji. The translators used literal translations of the ethnic foods via Google Translate and kept the English spelling of the food in parentheses.

Differences in semantics: Six of the questions contained examples of foods that have different meanings in English but have the same meaning in Chinese. For example, one question asked about green leafy salad or lettuce salad, all of which have the same meaning in Chinese. The translators provided literal translations of each word with an example for each – for example, spinach was an example of a green leafy salad.

### Comparison of ASA-24 data and ADSQ scores

Table 2 presents means and standard deviations of component scores for seven ADSQ components, means and standard deviations of these components as calculated from the ASA-24, and paired T-test analyses. A healthy diet includes high amounts of all listed components except for added sugars. Total vegetables (cups/day) and added sugars (tsp/day) were significantly different.

**Table 2:** Comparison of mean daily intake values, Adapted Dietary Screening Questionnaire versus Automated Self-Administered Dietary Assessment Tool-24 in the Validating a Dietary Assessment study, 2021

Component	ADSQ Mean SD		ASA-24 Mean SD		Mean Difference Mean SD		P-value
Total Vegetables (cups/day)	1.83	0.56	2.78	1.88	0.95	1.83	0.006
Total Fruit (cups/day)	1.00	0.37	1.02	0.96	0.02	0.99	0.90
Dairy (cups/day)	1.49	0.43	1.23	1.56	-0.26	1.62	0.36
Added Sugar (tsp/day)	13.96	3.62	8.60	9.62	-5.35	10.45	0.006
Fiber (g/day)	17.65	4.30	20.10	11.66	2.44	12.76	0.28
Calcium (mg/day)	943.90	157.48	797.71	467.10	-146.19	503.45	0.11
Whole Grains (oz/day)	0.70	0.40	0.67	0.86	-0.03	0.88	0.86

**Fig. 2:**
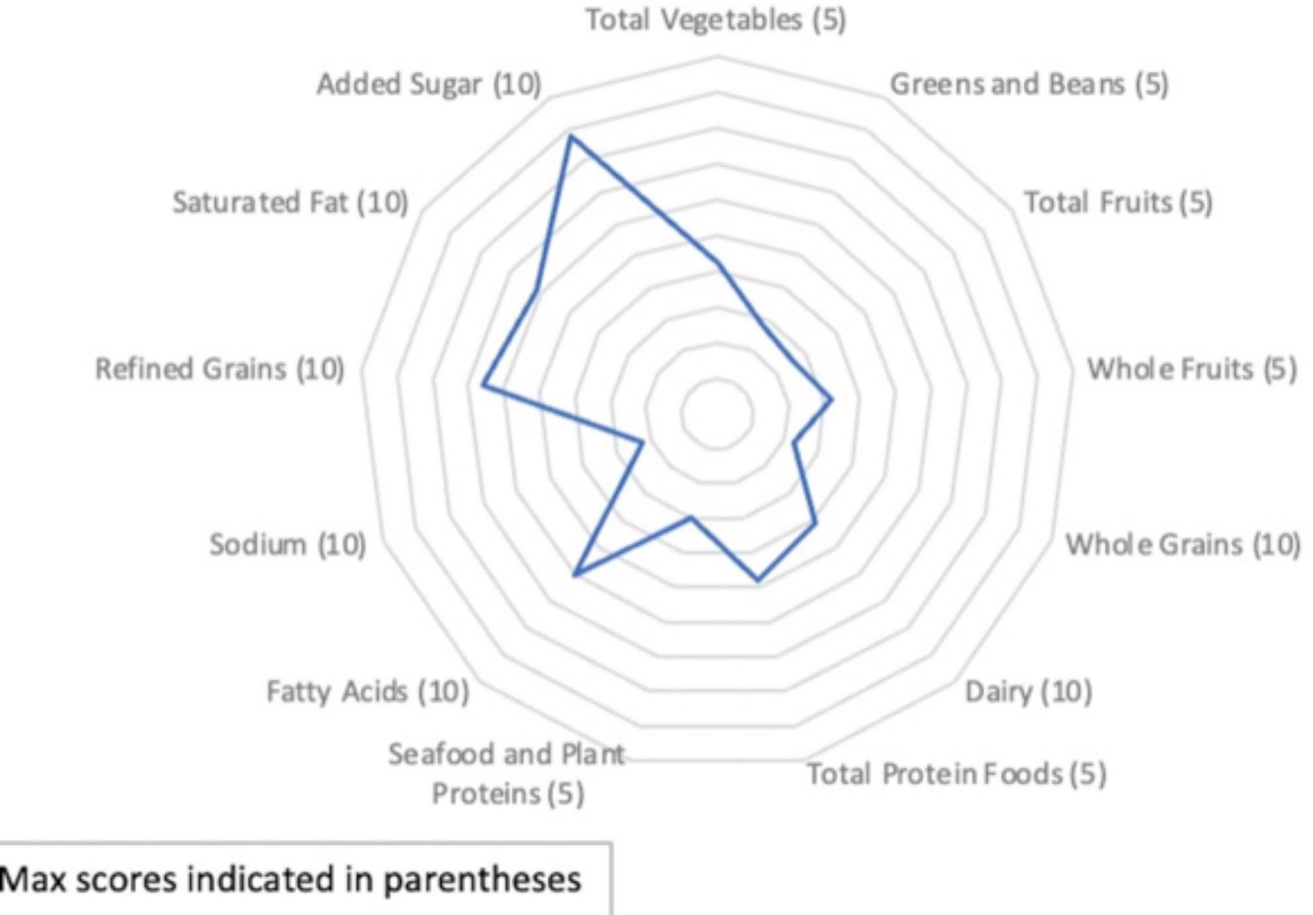
Healthy Eating Index (HEI) Component Scores in the Validating a Dietary Assessment study, 2021

HEI component scores: Mean component scores are plotted (Fig. 2) and maximum HEI scores reflect a diet quality that aligns with the 2015 Dietary Guidelines for Americans and would line the perimeter of a radar plot. Component scores closest to the center of the radar plot are those that could most use improvement, as they reflect categories whose actual score and maximum possible score are most discrepant. Highly discrepant categories include sodium, whole grains, total fruit, and dairy.

Correlation between ADSQ and ASA-24 data: Table 3 presents Spearman correlation coefficients between seven ADSQ and ASA-24 component values. All correlations were < 0.5, and there were no significant associations.

**Table 3:** Spearman correlations between Automated Self-Administered Dietary Assessment Tool-24 and Adapted Dietary Screening Questionnaire Values in the Validating a Dietary Assessment study, 2021

Component	Spearman’s Correlation Coefficient	Significance (2-tailed)
Total Vegetables (cups/day)	0.18	0.32
Total Fruit (cups/day)	0.05	0.77
Dairy (cups/day)	-0.13	0.48
Added Sugar (tsp/day)	-0.18	0.31
Fiber (g/day)	-0.06	0.73
Calcium (mg/day)	-0.09	0.63
Whole Grains (oz/day)	0.27	0.13

## Discussion

To the best of our knowledge, this is the first study to establish the criterion validity of a dietary screener specifically adapted for Asian Americans. In comparing our seven categories of nutrient consumption, paired T-tests identified total vegetables and added sugars as statistically significantly different between the ADSQ and ASA-24, suggesting that consumption data of these two components were most dissimilar. Similar to our findings, Hewawitharana et al. found that mean added sugar intake as reported from the original, un-adapted DSQ was significantly higher than that reported by the ASA24-Kids (19 vs. 12 tsp/day, p < 0.01) among 4–15 year olds sampled from 130 communities throughout the United States [24]. These findings suggest researchers use caution when using the DSQ, whether adapted or not, to estimate absolute intake of total vegetables and added sugars.

In correlating nutrient consumption between the ADSQ and ASA-24 components, correlations were moderate (r = 0.27) for whole grains but poor (r < 0.2) for the remaining nutrient categories. A previous study that compared food intake of Chinese women as reported by an adapted food frequency questionnaire (FFQ) compared to three 24-hour recalls [20] similarly found moderate-weak correlations. They found that for nutrient estimates, correlations were high (r > 0.5) for dietary fiber and calcium, whereas correlations were moderate or low for the remaining eleven nutrient categories. They posit that increasing the number of 24-hour recalls may improve agreeability, since a few individual records may not be reflective of overall diet as captured by the FFQ. In addition, they suggest identifying important foods, grouping them based on consumption and nutrient profile similarities, and then selecting nutrient values for the food groups [20]. Thus, it is possible that our ADSQ food groupings and/or nutrient scoring methods could be improved to more accurately portray CHA consumption.

Even further, it is challenging to estimate the intake of certain components that are comprised of a variety of foods. Highest correlations were between added sugars from sugar-sweetened beverages (SSBs) and fruits and vegetables. Lowest correlations were between total added sugars, whole grains, and dairy. The authors reason that this may be because it is easier to estimate intake of specific items (e.g., SSB) as opposed to a wide array of items that comprise one larger category (e.g., aspects of diet that constitute dairy). This may explain why our measured amounts of added sugar are significantly different between the ADSQ data and the ASA-24.

Using the ASA-24 to collect food record data presented some notable challenges. Although the ADSQ was adapted to include ethnic Asian foods, the ASA-24 does not include many ethnic foods. This limited our ability to fully capture dietary intake using the ASA-24 and may explain some discrepancies between food record and ADSQ component scores. Furthermore, our small sample size provided insufficient power to detect correlations < 0.5.

Finally, in analyzing difficulties in the translation from English to Simplified Chinese, we identified two main challenges: (1) translating non-Chinese ethnic foods into Simplified Chinese and (2) differences in semantics between English and Chinese that make it difficult to convey the exact meaning of the ADSQ to those who only speak Chinese. Future work is needed to improve approaches for asking non-English speaking participants about their consumption of ethnic foods that cannot be translated, as well as learning ways to maintain the semantic and literal meaning of an English sentence when translating it into Chinese.

Dietary screeners can be useful in assessing specific aspects of a diet of interest. To our knowledge, this adapted DSQ is the only dietary screener tailored to Asian Americans. We acknowledge that a single screener cannot capture the diversity of diets across Southeast Asia, South Asia, and Northeastern Asia [25]. The only other dietary screener tailored to a westernized-Asian population is a thirty-seven question dietary screener developed in Singapore and tested on a cohort of Chinese, Malay, and Indian-Singaporean participants, whose criterion validity was established by comparison to an FFQ [26]. Further development of the use and scoring of this ADSQ is vital in understanding the diets of CHA’s and other Asian subgroup populations in the US [25].

To evaluate the practicality of the ADSQ in clinical use, future studies could compare results from the ADSQ with common metabolic measures, such as total cholesterol and BMI, in order to identify food groups associated with different levels of these metabolic measures. One study found that participants with healthier diets had many improved health metrics, including lower BMI and lower cholesterol [27]. Another found that normal weight children had increased fruit and vegetable intake compared to overweight or obese children, whereas obese children had greater intake of total added sugars then overweight or normal children [24].

## Conclusion

The ADSQ may be useful in assessing specific aspects of dietary intake and, subsequently, areas of diet in most need of improvement. In this study, nutrient values obtained by the ADSQ were similar to those obtained by the ASA-24, with the exception of total vegetables and added sugars. In addition, both the ASA-24 and ADSQ identified the same components where CHA diet was poorest. Compared to more common methods, such as 24-hour recalls, food records and food frequency questionnaires (FFQs), DSQs are less resource-intensive and time-consuming. However, they do require the participant to calculate their usual intake of various foods, which may be more difficult and therefore less accurate than recording what they ate in the previous 24 h [24]. Our results suggest that the ADSQ may be useful in identifying unhealthy aspects of CHA diet. Future studies should focus on additional studies in the CHA population in order to identify additional strengths and weaknesses of the ADSQ to identify individuals who could benefit from dietary intervention to improve health outcomes.

## Data Availability

The datasets used and/or analyzed during the current study are available from the corresponding author on reasonable request.

## Abbreviations

ADSQ: Adapted Dietary Screener Questionnaire
ASA-24: Automated Self-Administered 24-Hour Dietary Assessment Tool
NHANES: National Health and Nutrition Examination Survey
CHW: Chinese American Adult (CHA), community health worker
NCI: National Cancer Institute
HEI: Health Eating Index
FFQ: Food frequency questionnaire
